# Vestibular hair cells are more prone to damage by excessive acceleration insult in the mouse with KCNQ4 dysfunction

**DOI:** 10.1038/s41598-024-66115-9

**Published:** 2024-07-03

**Authors:** Hansol Hong, Eun Ji Koo, Yesai Park, Gabae Song, Sun Young Joo, Jung Ah Kim, Heon Yung Gee, Jinsei Jung, Kangyoon Park, Gyu Cheol Han, Jae Young Choie, Sung Huhn Kim

**Affiliations:** 1 Department of Otorhinolaryngology, Won-Sang Lee Institute for Hearing Loss, Yonsei University College of Medicine and Graduate School of Medicine, Seoul, Republic of Korea; 2 Department of Otorhinolaryngology, Gachon University College of Medicine, Incheon, Republic of Korea; 3 Department of Otorhinolaryngology, Incheon St. Mary’s Hospital, Catholic University College of Medicine, Incheon, Republic of Korea; 4 Department of Pharmacology, Graduate School of Medical Science, Brain Korea 21 Project, Yonsei University College of Medicine, Seoul, Republic of Korea

**Keywords:** Cellular neuroscience, Ion channels in the nervous system, Oculomotor system, Sensory processing

## Abstract

KCNQ4 is a voltage-gated K^+^ channel was reported to distribute over the basolateral surface of type 1 vestibular hair cell and/or inner surface of calyx and heminode of the vestibular nerve connected to the type 1 vestibular hair cells of the inner ear. However, the precise localization of KCNQ4 is still controversial and little is known about the vestibular phenotypes caused by KCNQ4 dysfunction or the specific role of KCNQ4 in the vestibular organs. To investigate the role of KCNQ4 in the vestibular organ, 6-*g* hypergravity stimulation for 24 h, which represents excessive mechanical stimulation of the sensory epithelium, was applied to p.W277S *Kcnq4* transgenic mice. KCNQ4 was detected on the inner surface of calyx of the vestibular afferent in transmission electron microscope images with immunogold labelling. Vestibular function decrease was more severe in the *Kcnq4*^p.W277S/p.W277S^ mice than in the *Kcnq4*^+/+^ and *Kcnq4*^+/p.W277S^ mice after the stimulation. The vestibular function loss was resulted from the loss of type 1 vestibular hair cells, which was possibly caused by increased depolarization duration. Retigabine, a KCNQ activator, prevented hypergravity-induced vestibular dysfunction and hair cell loss. Patients with *KCNQ4* mutations also showed abnormal clinical vestibular function tests. These findings suggest that KCNQ4 plays an essential role in calyx and afferent of type 1 vestibular hair cell preserving vestibular function against excessive mechanical stimulation.

## Introduction

The inner ear comprises the cochlea and the vestibule, which detect sound pressure and acceleration, respectively. The vestibule consists of the utricle, the saccule, and three semicircular canals. The utricle and saccule detect linear acceleration, and the semicircular canals detect angular acceleration; this sensory input is used to maintain balance in daily activities. The sensory transduction of these mechanical stimuli is regulated by various ion channels, transporters, and exchangers distributed over the epithelial cells of inner ear^1^. Among these ion channels, K^+^ channels in the basolateral surface of sensory hair cells in the cochlea and vestibule take part in the repolarization of hair cells by providing a means of efflux for intracellular K^+^ ions that have entered to the hair cells through mechanosensitive nonselective cation channels at the stereocilia in response to mechanical stimulation^1–3^. If the process is not properly maintained, hearing loss, dizziness and imbalance occur. One of the main K^+^ channels involved in the repolarization of cochlear outer hair cells is KCNQ4, a voltage-gated K^+^ channel that belongs to the Kv7 (KCNQ) family^3^. In humans, mutations in *KCNQ4* cause nonsyndromic sensorineural hearing loss with an autosomal dominant inheritance pattern, and this hearing loss is aggravated after noise exposure^4–11^. The mechanism of hearing loss in KCNQ4 dysfunction has not been definitively identified, but it is thought that hair cell injury results from repolarization failure^12^. Although KCNQ4 is also found in the vestibule, the location of distribution is still controversial and regarded as different from the distribution location at the cochlear outer hair cells. It was found to be located at the basolateral membrane of type 1 and/or type 2 vestibular hair cells, and/or the inner surface of the calyx membrane that faces the basolateral surface of type 1 vestibular hair cells, and the heminode of the calyx-only afferent nerve in animal experiments^13–19^. In the studies, KCNQ4-mediated currents have been detected from the vestibular hair cells during development, but their expression declines substantially with maturation. Although there are many animal and human studies on the relationship between hearing loss and KCNQ4 dysfunction^12^, the relationship between KCNQ4 dysfunction and vestibular disorder has not been definitively identified.

We hypothesized that mutation of *Kcnq4* may cause vestibular dysfunction after excessive mechanical stimulation due to the failure of [K^+^] regulation in the type 1 vestibular hair cell cytosol, similar to the mechanism of noise-induced hearing loss due to *Kcnq4* mutation in mouse model. We developed a new experimental method of hypergravity challenge in mice, which involves excessive mechanical stimulation of the vestibular organ similar to loud sound stimulation of the cochlea. We applied this method to investigate the mechanism of vestibular dysfunction in p.W277S/p.W277S *Kcnq4* transgenic mice. We selected the p.W277S mutation for the experiment because the mutation corresponds to p.W276S mutation in human (both mutation is caused by c.830G > C mutation in exon 5) and is one of the mutations with the severest cochlear phenotype in humans^20^. We expected that the mice with the mutation would also have a more severe vestibular phenotype than the other mutations because the molecular structures of certain cellular components such as vestibular and cochlear hair cells, vestibular and cochlear supporting cells, and vestibular dark cells and strial marginal cells in cochlea, are homologous in both systems. It has been reported that patients with p.W276S *KCNQ4* mutations showed hyperactive or hypoactive vestibulo-ocular reflex (VOR) to roatary and/or caloric stimulation^21^. These studies used caloric tests and/or rotary chair tests for the evaluation. Although those tests are gold standards in evaluating vestibular function in humans, there are several limitations. The caloric test can evaluate the response of each lateral semicircular canal, but it is difficult to identify bilateral partial vestibular loss because the test result is interpreted by comparing the response of the right and left sides. The results of the rotary chair test can be nearly normal in patients with centrally compensated chronic bilateral partial vestibular loss. Therefore, there are still controversies about the vestibular phenotype of patients with the p.W276S mutation.

In this study, functional and histological changes in vestibular organs after stimulation challenge was examined using animal vestibular function tests and molecular biological methods. We also investigated the precise location of KCNQ4 in type 1 vestibular hair cells using transmission electron microscopy (TEM) with immunogold staining. We investigated the vestibular phenotype of patients with *KCNQ4* mutations. We adopted video head impulse test (vHIT), a recently developed tool for evaluating all the functions of the semicircular canals over a range of high-frequency stimuli, that is useful for evaluating the vestibular function in those cases and cervical/ocular vestibular evoked myogenic potentials (cVEMP/oVEMP) for the evaluation of otolithic organs. We believe that the results of this study can provide a basis for the identification of the pathological mechanism of balance disorder caused by KCNQ4 dysfunction as well as physiological role of KCNQ4 in the peripheral vestibular system in the maintenance of balance.

## Results

### Changes in vestibular function after hypergravity stimulation

**Figure 1:**
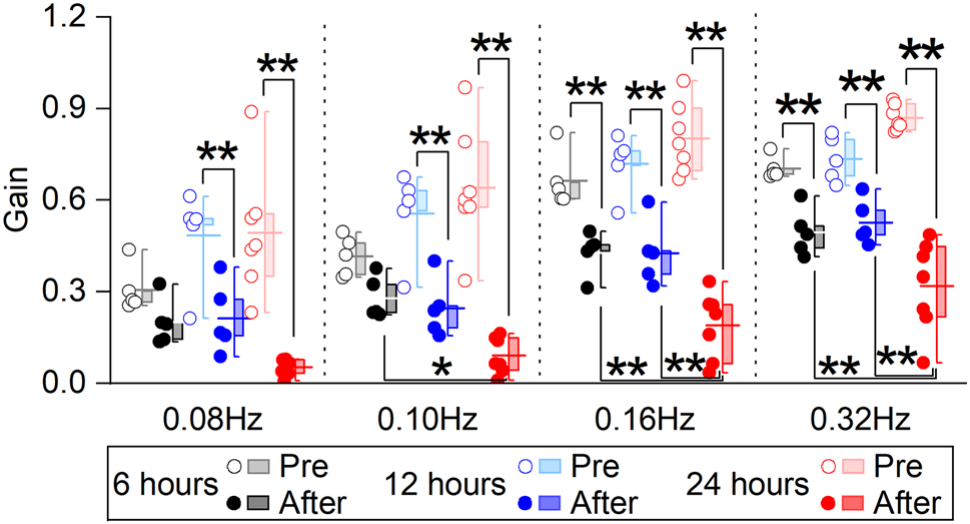
Time-dependent changes in the vestibulo-ocular reflex (VOR) gain after 6-*g* hypergravity stimulation in *Kcnq4* ^p.W277S/p.W277S^ mice. There was no significant difference between the gain values after 6 h and 12 h of stimulation, but gain values were significantly lower after 24 h of stimulation than after 6 or 12 h of stimulation (n = 5 for each condition). *, *p* < 0.05 ; **, *p* < 0.01 (two-way repeated-measures ANOVA with Holm-Sidak posttest); box, 25–75%; horizontal line in the box, mean; whisker, range within 1.5 times the interquartile range; pre, before hypergravity stimulation; after, after hypergravity stimulation.

First, we applied 6-*g* hypergravity stimulation to *Kcnq4* ^p.W277S/p.W277S^ mice for 6-24 hours as a pilot study. Then, we compared the difference in the VOR gain in the slow harmonic acceleration (SHA) test, which mainly represents the function of the lateral semicircular canal, between pre- and post-stimulation to determine the appropriate stimulation duration for the whole experiment. The VOR gain started to decline from the stimulation duration of 6 hours at 0.16 and 0.32Hz (Fig. 1 and Supplementary Data S1). The VOR gains significantly decreased after 12 and 24 hours of stimulation throughout the frequency range (Fig. 1 and Supplementary Data S1). The poststimulation gain values were not significantly different between the mice treated with 6 and 12 hours of stimulation throughout the frequency range (Fig. 1 and Supplementary Data S1). However, 24 hours of stimulation resulted in a significantly lower gain than 6 and 12 hours of stimulation at 0.10, 0.16 and 0.32 Hz (Fig. 1 and Supplementary Data S1). Therefore, we decided to use 24 hours of stimulation to evaluate the differences in the VOR parameters among the genotypes.

**Figure 2:**
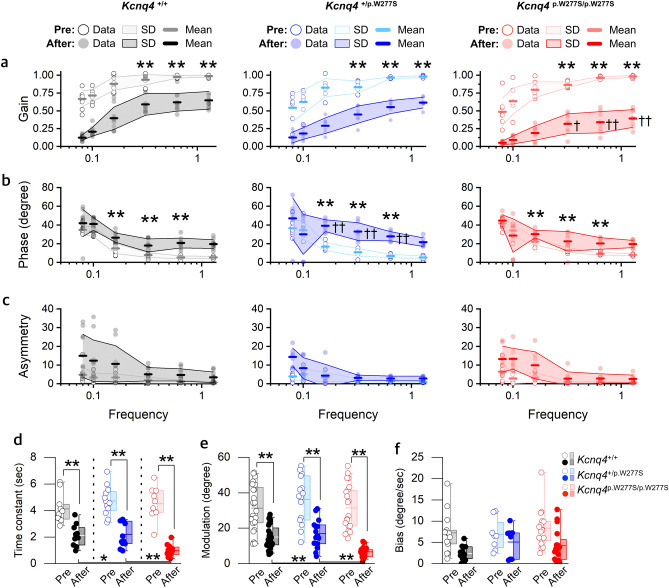
Differences in the parameters of the vestibulo-ocular reflex among the *Kcnq4* ^+/+^, *Kcnq4* ^+/p.W277S^, and *Kcnq4* ^p.W277S/p.W277S^ mice after 6-*g* hypergravity stimulation for 24 h. (**a**) – (**c**). Gain (**a**), phase (**b**), and asymmetry (**c**) in the slow harmonic acceleration test (n = 11, 7, and 8 for *Kcnq4* ^+/+^, *Kcnq4* ^+/p.W277S^, and *Kcnq4* ^p.W277S/p.W277S^ mice). (**d**) Time constant in the step velocity test (n = 6, 5, and 6 for *Kcnq4* ^+/+^, *Kcnq4* ^+/p.W277S^, and *Kcnq4* ^p.W277S/p.W277S^ mice). (**e**) and (**f**). Modulation (e, n = 11, 14, and 8 for *Kcnq4* ^+/+^, *Kcnq4* ^+/p.W277S^, and *Kcnq4* ^p.W277S/p.W277S^ mice) and bias (f, n = 8, 4, and 8 for *Kcnq4* ^+/+^, *Kcnq4* ^+/p.W277S^, and *Kcnq4* ^p.W277S/p.W277S^ mice) in off-vertical axis rotation test. Note that gain values at 0.32 – 1.28 Hz were significantly decreased and phase differences at 0.16—0.64 Hz were significantly increased after stimulation in all genotypes ((**a**)–(**c**), **). The *Kcnq4* ^p.W277S/p.W277S^ mice exhibited the lowest gain value at 0.32 – 1.28 Hz ((**a**), ┼, lower gain in *Kcnq4* ^p.W277S/p.W277S^ than those in *Kcnq4* ^+/+^ and *Kcnq4* ^+/p.W277S^ mice, and lower gain in *Kcnq4* ^+/p.W277S^ than that in *Kcnq4* ^+/+^ mice; ┼┼, lower gain in *Kcnq4* ^p.W277S/p.W277S^ than those in *Kcnq4* ^+/+^, *Kcnq4* ^+/p.W277S^ mice; (**b**), ┼┼, higher phase in *Kcnq4* ^+/p.W277S^ than those in *Kcnq4* ^+/+^, *Kcnq4* ^p.W277S/p.W277S^ mice ), time constant (**d**) and modulation (**e**) after stimulation compared with those of the *Kcnq4* ^+/+^ and *Kcnq4* ^+/p.W277S^ mice. *, *p* < 0.05; **, *p* < 0.01; ┼ and ┼┼, *p* < 0.05 (inter-genotype comparison in (**a**)–(**c**)) (two-way repeated-measures ANOVA with Holm-Sidak posttest); box, 25–75%; horizontal line in the box, mean; whisker, range within 1.5 interquartile range; pre, before hypergravity stimulation; after, after hypergravity stimulation. The mean phase value for the *Kcnq4*^p.W277S/p.W277S^ mice was higher than that for the *Kcnq4* ^+/+^ mice before stimulation, but it was not annotated with asterisks because the phase values of those mice were within the normal range.

The parameters (gain, phase, symmetry) of the SHA test among *Kcnq4*^+/+^, *Kcnq4*^+/p.W277S^, and *Kcnq4*^p.W277S/p.W277S^ mice before 6-*g* stimulation were not significantly different at all frequencies, except phase at 0.16 Hz where the *Kcnq4*^p.+/p.W277S^ mice showed a higher phase value than *Kcnq4* ^+/+^ (Fig. 2a and Supplementary Data S2). We thought that the difference in the phase value at 0.16 Hz was not meaningful because the values were within the normal range. After 24 hours of stimulation, the gain values associated with all the mouse genotypes were significantly decreased at 0.32 – 1.28 Hz of SHA test (Fig. 2a and Supplementary Data S2). In particular, the gain values at 0.32 – 1.28 Hz after 6-*g* stimulation was significantly lower in the *Kcnq4*^p.W277S/p.W277S^ mice than in the *Kcnq4*^+/+^ and *Kcnq4*^+/p.W277S^ mice (Fig. 2a and Supplementary Data S2). *Kcnq4*^+/p.W277S^ mice showed lower gain value only at 0.32 Hz than *Kcnq4*^+/+^ mice.

The phase difference was also increased in all the genotypes at 0.16 and 0.64 Hz after stimulation, and the increase was largest in the *Kcnq4*^+/p.W277S^ mice (Fig. 2b and Supplementary Data S2). Symmetry was not different among the genotypes before and after stimulation (Fig. 2c and Supplementary Data S2). In step velocity (SV) test, which represents lateral semicircular canal function in higher frequency ranges of stimulation, the time constant (Tc) was significantly decreased after stimulation in all the genotypes (Fig. 2d and Supplementary Data S2), and it was significantly smaller in the *Kcnq4*^p.W277S/p.W277S^ mice than in the *Kcnq4*^+/+^ and *Kcnq4*^+/p.W277S^ mice (Fig. 2d and Supplementary Data S2). In off-vertical axis rotation (OVAR) test, which represents the function of otolithic organs, the modulation of the VOR was significantly decreased after stimulation in all the genotypes (Fig. 2e and Supplementary Data S2). It was also significantly lower in the *Kcnq4*^p.W277S/p.W277S^ mice than in the *Kcnq4*^+/+^ and *Kcnq4*^+/p.W277S^ mice (Fig. 2e and Supplementary Data S2). Changes in the bias value were not significant after stimulation (Fig. 2f and Supplementary Data S2).

### Changes in hair cells and KCNQ4-positive synaptic *calyx* nerve endings after hypergravity stimulation

**Figure 3:**
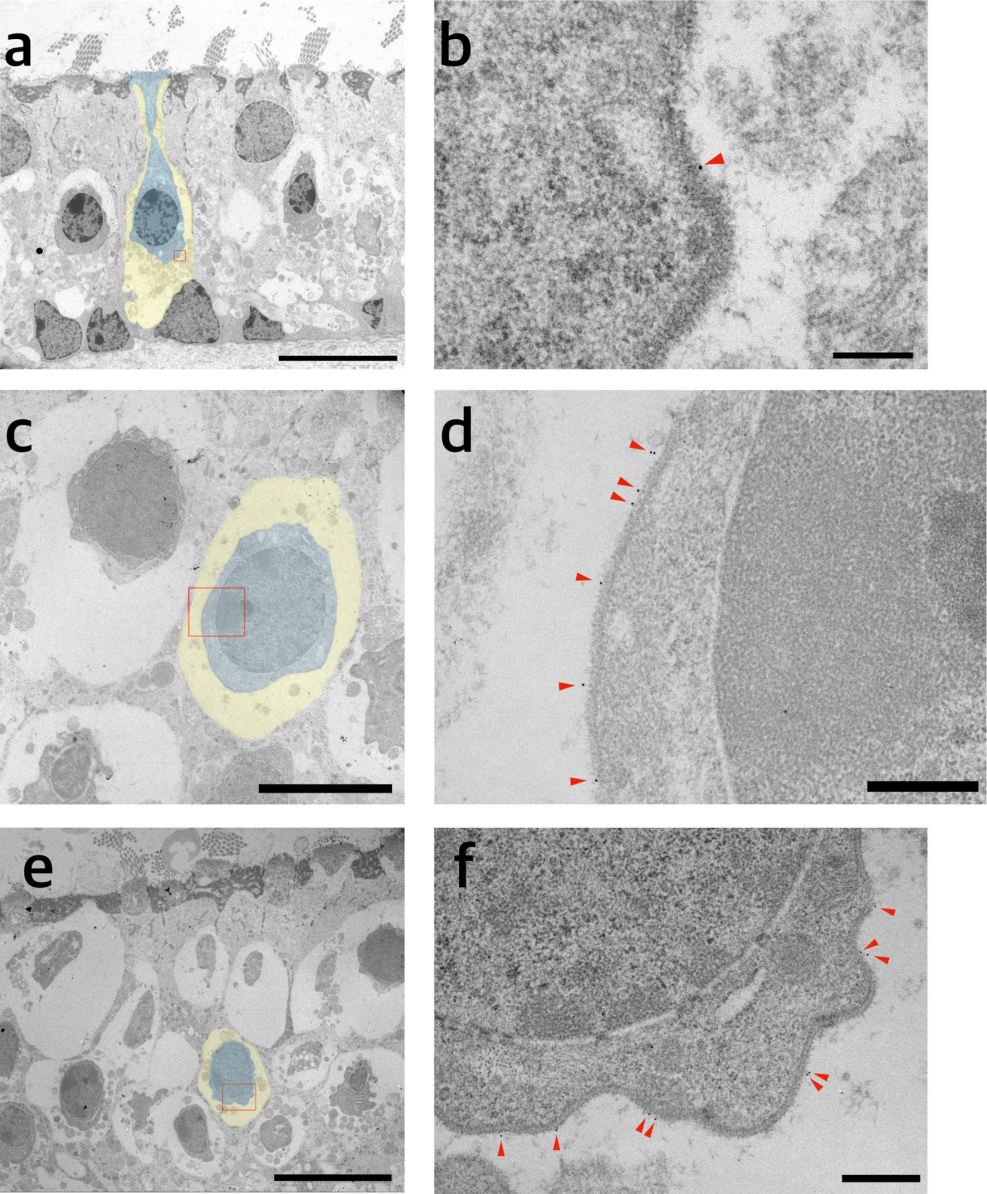
Representative transmission electromicroscopic images with immunogold labelling of KCNQ4 in utricular sensory epithelia. (**a**) Coronal section of utricular sensory epithelium (scale bar = 10 µm). (**b**) Magnified view of basolateral memrane of type 1 vestibular hair cell and calyx (rectangular area of (**a**)) (Scale bar = 200 nm). (**c**)–(**f**) Oblique axial section of utricular sensory epithelium (scale bar = 5 µm in (**c**); 500 nm in (**d**); 10 µm in (**e**); 500 nm in (**f**)). Blue area, type 1 vestibular hair cell; yellow area, calyx of vestibular afferent nerve in (**a**), (**c**) and (**e**). Red arrow indicates immunogold labelled KCNQ4 in (**b**), (**d**), (**f**).

The distribution of KCNQ4 in the vestibular sensory epithelium could be detected in the vestibular calyx and/or basolateral surface of type 1 vestibular hair cell, and vestibular nerve as previously reported, and the expression overlapped with anti-calretinin staining (Supplementary Fig. S1)^15^. To examine if the distribution of KNCQ4 was on the basolateral surface of type 1 vestibular hair cells or the inner surface of calyx or both surface, TEM with immunogold labelling of KCNQ4 was performed. In TEM imaging, KCNQ4 was only detected on the inner surface of calyx of type 1 vestibular hair cell (Fig 3).

**Figure 4:**
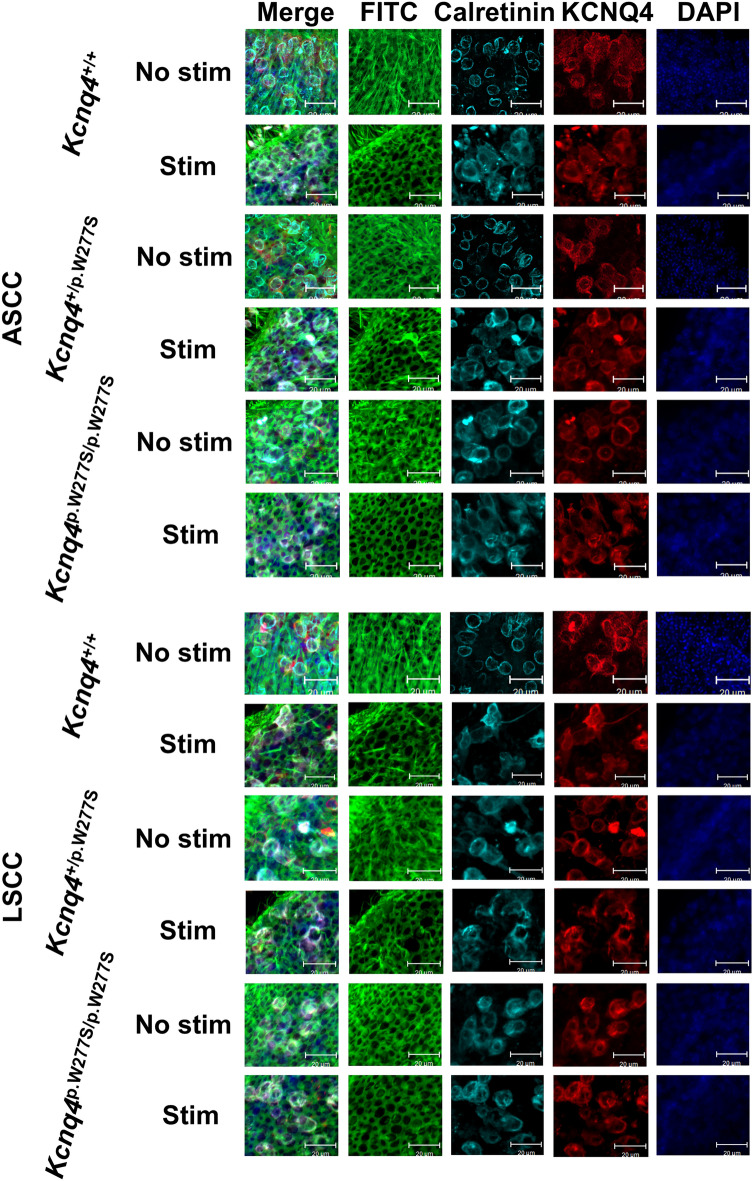
Representative images of immunofluorescent staining of the anterior semicircular canal (ASCC) and lateral semicircular canal (LSCC) from the *Kcnq4* ^+/+^, *Kcnq4* ^+/p.W277S^ and *Kcnq4* ^p.W277S/p.W277S^ mice with or without stimulation. Hair cells with cilia loss were evident after stimulation in the ampulary crest of all genotypes (FITC) when compared to hair cells in the ampullary crest of mice without stimulation. In merged images (Merge), calretinin/KCNQ4-positivie hair cells with intact cilia in the ampullary crest without stimulation were mostly intact, however, those in the ampullary crest after stimulation were rare. Especially, this finding was more significant in the ampullary crest of *Kcnq4* ^p.W277S/p.W277S^ mice (scale bar = 20 μm). No stim, no stimulation; Stim, after stimulation.

**Figure 5:**
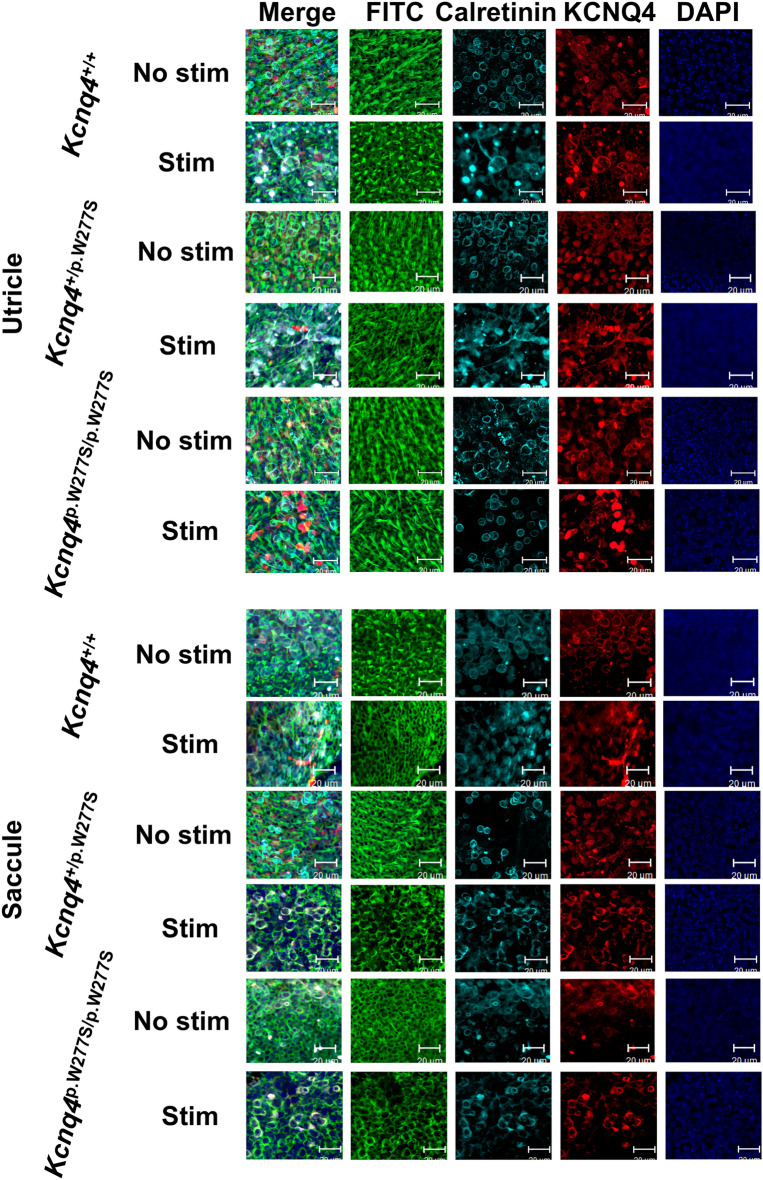
Representative images of immunofluorescent staining of utricle and saccule from the *Kcnq4* ^+/+^, *Kcnq4* ^+/p.W277S^ and *Kcnq4* ^p.W277S/p.W277S^ mice with or without stimulation Hair cells with cilia loss were evident after stimulation in the saccular macula of all genotypes (FITC) when compared to hair cells in the saccular macula of mice without stimulation. In merged images (Merge), calretinin/KCNQ4-positivie hair cells with intact cilia in the saccular macula of mice without stimulation were mostly intact, however, those in the saccular macula of mice after stimulation were rare. Especially, this finding was more significant in the macula of *Kcnq4* ^p.W277S/p.W277S^ mice (scale bar = 20 μm). No stim, no stimulation; Stim, after stimulation.

**Figure 6:**
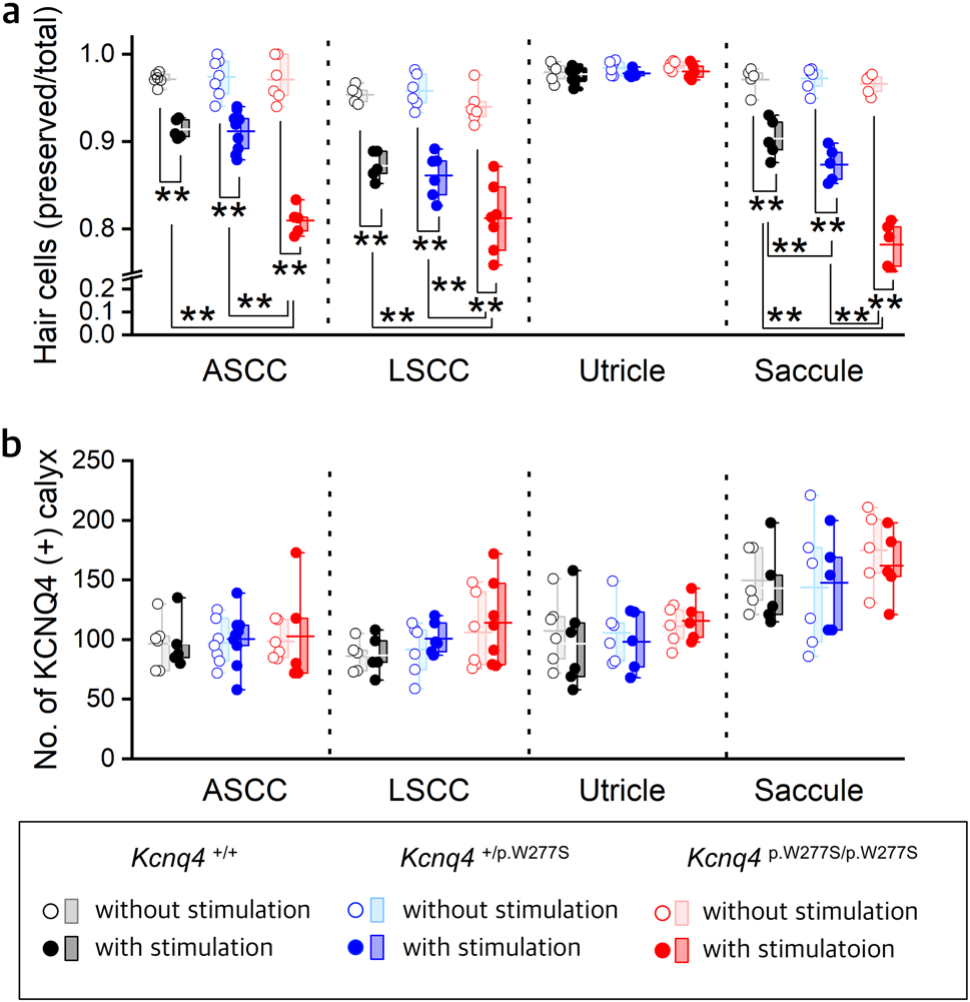
Differences in the ratio of intact hair cell to total hair cell count where KCNQ4 and calretinin-positive stain among the *Kcnq4* ^+/+^, *Kcnq4* ^+/p.W277S^, and *Kcnq4* ^p.W277S/p.W277S^ mice with or without hypergravity stimulation for 24 h. (**a**) and (**b**). Summary graph of the ratio of intact hair cell to total hair cell count (**a**) and the number of KCNQ4/calretinin-positive calyx nerve endings (KCNQ4 ( +) calyx) (**b**) with and without hypergravity stimulation for 24 h vestibular sensory epithelium in the anterior semicircular canal (ASCC), lateral semicircular canal (LSCC), utricle, and saccule of each mouse genotype (n of *Kcnq4* ^+/+^, *Kcnq4* ^+/p.W277S^, and *Kcnq4* ^p.W277S/p.W277S^ mice without stimulation / with stimulation; ASCC, 6, 7, and 6 / 5, 9, and 5; LSCC, 5, 6, and 6 / 5, 6, and 7; utricle, 5, 6, and 5 /6, 5, and 5; saccule, 5, 6, and 5 / 5, 5, and 5). The ratio of intact hair cell to total hair cell count in the ASCC, LSCC, and saccule significantly decreased in the *Kcnq4* ^p.W277S/p.W277S^ mice than in the *Kcnq4* ^+/+^ and *Kcnq4* ^+/p.W277S^ mice. In addition, the ratio of intact hair cell to total hair cell count in the saccule significantly decreased in the *Kcnq4* ^+/p.W277S^ mice than in the *Kcnq4* ^+/+^ mice. The number of calyx was not different among the genotypes. **, *p* < 0.01 (two-way ANOVA with Holm-Sidak posttest). Box, 25–75% range; horizontal line in the box, mean; whisker, range within 1.5 times the interquartile range.

Then, whole-mount immunofluorescent staining of vestibular sensory epithelium was performed to evaluate the microstructural changes after hypergravity stimulation. The number of KCNQ4-positive calyx and the ratio of intact hair cell to total KCNQ4 and calretinin stained hair cell count in the sensory epithelium of the utricle, lateral and anterior ampulla, and saccule of the mice without stimulation were not significantly different (Figs. 4–6 and Supplementary Data S3).

After stimulation, the number of KCNQ4-positive calyx was not changed, however, the ratio of intact hair cell to total KCNQ4 and calretinin stained hair cell count was significantly decreased in the anterior and lateral semicircular canal ampulla and the saccule of the *Kcnq4*^p.W277S/p.W277S^ mice than in the *Kcnq4*^+/+^ and *Kcnq4*^+/p.W277S^ mice (Fig. 6 and Supplementary Data S3) after stimulation. In the saccule, the ratio in the *Kcnq4*^+/p.W277S^ mice was significantly decreased than that in the *Kcnq4*^+/+^ mice (Fig. 6d and Supplementary Data S3). There was no difference in the ratio in the utricle among the genotypes after stimulation (Fig. 6c and Supplementary Data S3).

### Differences in the intracellular Ca^2+^ decay durations among the genotypes

**Figure 7:**
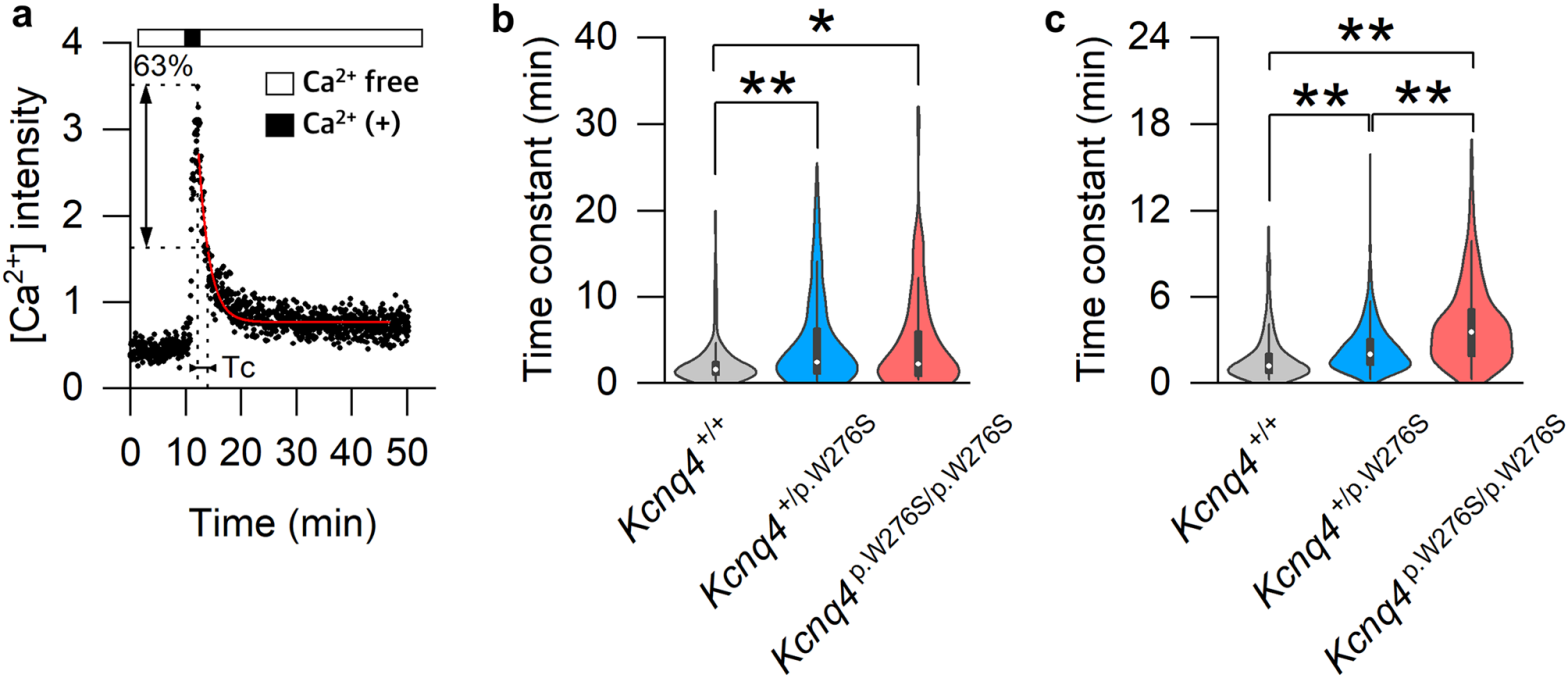
Differences in the time constant (Tc) for the decaying intracellular [Ca^2+^] signal from the sensory epithelium of the *Kcnq4* ^+/+^, *Kcnq4* ^+/p.W277S^, and *Kcnq4* ^p.W277S/p.W277S^ mice. (**a**) Representative figure for negative exponential curve fitting for [Ca^2+^] decay (red line) for the calculation of Tc. (**b**) Tc distribution in the ampullary crest (n of cells = 134, 91, and 96 for *Kcnq4* ^+/+^, *Kcnq4* ^+/p.W277S^, and *Kcnq4* ^p.W277S/p.W277S^ mice). (c) Tc distribution in the utricular macula (n of cells = 229, 549, and 159 for *Kcnq4* ^+/+^, *Kcnq4* ^+/p.W277S^, and *Kcnq4* ^p.W277S/p.W277S^ mice). The Tc order was *Kcnq4* ^p.W277S/p.W277S^ = *Kcnq4* ^+/p.W277S^ > *Kcnq4* ^+/+^ in the ampullary crest and *Kcnq4* ^p.W277S/p.W277S^ > *Kcnq4* ^+/p.W277S^ > *Kcnq4* ^+/+^ in the utricular macula. *, *p* < 0.05; **, *p* < 0.01 (one-way ANOVA on ranks with Dunn’s posttest).

We evaluated the Tc of the decaying intracellular [Ca^2+^] signal by live imaging to indirectly evaluate the duration of depolarization of cells in the sensory epithelium of the utricle and ampulla of each mouse genotype. After the perfusion of Ca^2+^-containing perilymph-like solution, the decaying intracellular [Ca^2+^] signal showed a negative exponential curve (Figure 7a). The Tc order for the negative exponential curve in the sensory epithelium of the ampulla was *Kcnq4*^p.W277S/p.W277S^ mice = *Kcnq4*^+/p.W277S^ mice > *Kcnq4*^+/+^ mice (Tc = 2.26 (0.81–6.11), 2.43 (1.12–6.36), and 1.57 (0.92–2.50), respectively; Figure 7b and Data S4), and the Tc order in the sensory epithelium of the utricle was *Kcnq4*^p.W277S/p.W277S^ mice > *Kcnq4*^+/p.W277S^ mice > *Kcnq4*^+/+^ mice (Tc = 3.57 (1.89–5.18), 2.02 (1.28–3.07), and 1.18 (0.67–2.07), respectively; Figure 7c and Data S4).

### Preventive effect of retigabine on vestibular dysfunction induced by hypergravity stimulation

We investigated the effect of retigabine, a KCNQ2-5 opener, on hypergravity-induced vestibular loss to provide further evidence that KCNQ4 dysfunction contributes to vestibular loss. We treated retigabine (10 μg/g) or DMSO as a control before stimulation and measured the VOR before and after stimulation in the *Kcnq4*^+/+^ mice. We used *Kcnq4*^+/+^ mice instead of *Kcnq4*^p.W277S/p.W277S^ mice for two reasons. First, retigabine is not effective on channels bearing the pore mutation p.W277S ^7^. Second, *Kcnq4*^+/+^ mice also had decreased VOR and vestibular hair cell lost after stimulation, and retigabine could have effect on intact KCNQ4 in *Kcnq4*^+/+^ mice. Retigabine was known to act on KCNQ4 as early as 2012 and confirmed to prolong the opening of KCNQ2 and KCNQ3, but it has also been demonstrated to increase the current from KCNQ4^7,22^. Because there is no currently available KCNQ4 selective activator, it was necessary to use retigabine for the supporting experiment.

**Figure 8:**
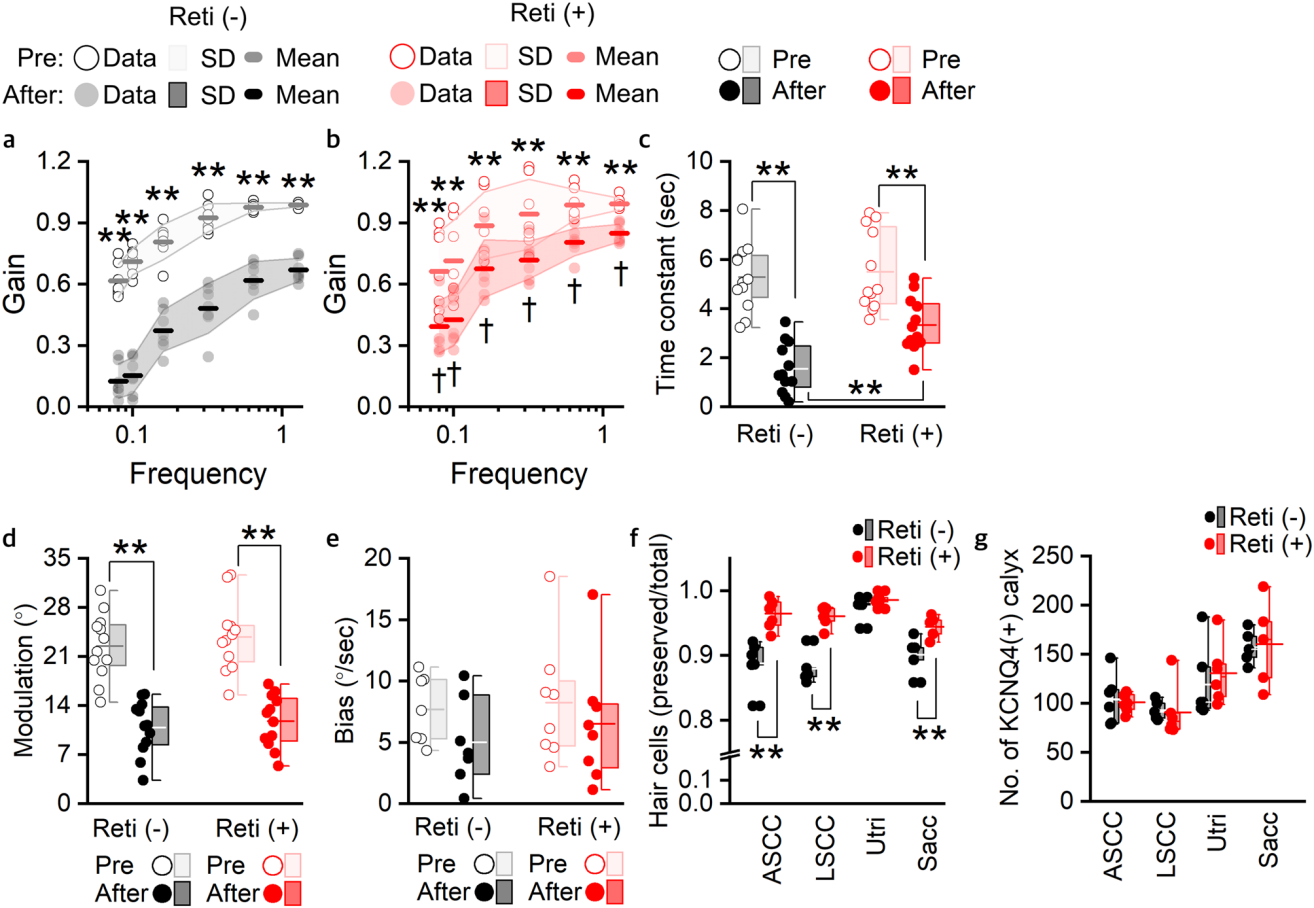
Differences in the results of vestibulo-ocular reflex in the rotation tests and the ratio of intact hair cell to total hair cell count where KCNQ4 and calretinin-positive stain between *Kcnq4* ^+/+^ mice treated with DMSO or retigabine after hypergravity stimulation. (**a**) Gain values in slow harmonic acceleration tests with DMSO (n = 7). (**b**) Gain values in slow harmonic acceleration tests with retigabine treatment (n = 7). (**c**) Time constants in step velocity tests (n = 6 for each group). (**d**) Modulation in off-vertical axis rotation (n = 6 for each group). (**e**) Bias in off-vertical axis rotation (n = 6 for each group). (**f**) the ratio of intact hair cell to total hair cell count where KCNQ4 and calretinin-positive stain (n of DMSO and retigabine-treated group; anterior semicircular canal, 6 and 7; lateral semicircular canal, 5 and 6; utricle , 6 and 6; saccule, 5 and 5). (**g**) The number of KCNQ4/calretinin-positive calyx nerve endings (KCNQ4 (+) calyx) (n of DMSO and retigabine-treated group is same with that in (**f**)). Gain values at all test frequencies and the time constants were significantly decreased after stimulation in DMSO and retigabine treated group ((**a**)–(**c**), **), but the gain values after stimulation were significantly higher in the retigabine-treated mice than those in DMSO-treated mice ((**b**), ┼). Hair cells were significantly preserved after stimulation in the mice treated with retigabine compared to those treated with DMSO (**f**). **; *p* < 0.01; ┼, p < 0.05 (comparison between DMSO and retigabine-treated mice in A and B) (two-way repeated-measures ANOVA with Holm-Sidak posttest for (**a**)–(**e**); t-test or Mann–Whitney rank sum test for (**f**) and (**g**)); In (**a**) and (**b**), circle, shaded area, and horizontal bar indicate data, SD, and mean. In (**c**)–(**g**), box, 25–75% range; horizontal line in the box, median; cross, mean; whisker, range within 1.5 times the interquartile range. Reti (−), DMSO treated; Reti ( +), retigabine treated. In (**f**) and (**g**), ASCC, anterior semicircular canal; LSCC, lateral semicircular canal; Utri, utricle; Sacc, saccule.

The gain, Tc, modulation, and bias before stimulation were not different between the mice treated with retigabine and those treated with DMSO (Fig. 8a–e and Supplementary Data S5). Although the gain values were significantly decreased in all test frequencies in both DMSO and retigabine groups, the gain values throughout the stimulation range (Fig. 8a, b and Supplementary Data S5) and Tc after stimulation (Fig. 8c and Supplementary Data S5) were revealed to be significantly preserved in the mice treated with retigabine compared with those treated with DMSO. Modulation was significantly decreased in both groups after stimulation, but the difference after stimulation was not significant (Fig. 8d and Supplementary Data S5). Bias was not significantly changed after stimulation in either group (Fig. 8e and Supplementary Data S5). Immunofluorescent staining of the vestibular sensory epithelium indicated that the ratio of intact hair cell was significantly lower in the mice treated with DMSO than in the mice treated with retigabine in the anterior and lateral semicircular canal ampulla and saccule (Fig. 8f and Supplementary Data S5). There was no difference in the number of KCNQ4-positive calyx between DMSO- and retigabine-treated mice (Fig 8g). Although we could not completely exclude the possibility of retigabine effect on KCNQ2 and KCNQ3, its effect on the KCNQ4 of vestibular neuron should also contribute to the preservation of vestibular function during stimulation.

### Vestibular phenotype of patients with KCNQ4 mutation

We investigated the vestibular function of patients (n = 10) with *KCNQ4* mutations in our genetic hearing loss cohort by clinical vestibular function test including the vHIT for semicircular canals and the cVEMP and oVEMP for otolithic organs. All the patients had variable degrees of progressive sensorineural hearing loss with an autosomal dominant inheritance pattern (Table 1). The patients with at least one abnormal result in vHIT, cVEMP and oVEMP accounted for 70.0% (7/10) of the total population (Table 1). The most common phenotype was loss of oVEMP (n = 5), followed by cVEMP (n = 4) and vHIT (n = 4) (Table 1 and Supplementary Fig. S2), which indicates that the mutation can affect either otolithic organs or semicircular canal function. Although the enrolled patients did not have the p.W276S mutation, the mutations in the patients caused hereditary sensorineural hearing loss. They had various degrees of KCNQ4 dysfunction, and we believe that the human data used in this study can represent the vestibular phenotype of patients with KCNQ4 mutations.

**Table 1:** Information about the patients with KCNQ4 mutations.

Patient no	Age	Sex	Mutation	Hearing loss onset	Pure tone threshold^†^ (Rt/Lt dBHL)	Pure tone audiogram pattern	vHIT	oVEMP	cVEMP
1	23	F	p.V87_N89del (c.259_267del), heterozygote	10 Y.O	39/45	Ski slope	NL	NL	NL
2	55	M	p.V87_N89del (c.259_267del), heterozygote	~ 20 Y.O	46/50	Ski slope	NL	Both loss	NL
3	25	F	p.V87_N89del (c.259_267del), heterozygote	Not sure	8/10	High tone loss (6 and 8kHz)	NL	Both loss	NL
4	41	M	p.L47P (c.140 T > C), heterozygote	18 Y.O	100/102	Down slope	NL	N/A	Rt. loss
5	22	M	p.L47P (c.140 T > C),, heterozygote	16 Y.O	76/73	Flat	Lt ASCC AbNL	N/A	Rt. loss
6	63	M	p.L47P (c.140 T > C), heterozygote	Not sure	38/18	High tone loss (4, 6, and 8kHz)	Rt LSCC AbNL	Both loss	Both loss
7	20	M	p.L47P (c.140 T > C), heterozygote	17 Y.O	64/13	Rt ski slope/Lt. high tone loss (6 and 8kHz)	Rt PSCC AbNL	Rt. loss	NL
8	7	M	p.G307V (c.920G > T), heterozygote	Several years	34/34	Down slope	NL	NL	NL
9	9	F	p.G307V (c.920G > T), heterozygote	Several years	41/39	Down slope	NL	NL	NL
10	36	F	p.G307V (c.920G > T), heterozygote	Child	102/100	Ski slope	Lt LSCC AbNL	Both loss	Both loss

Siblings, patients #1 – 3, #5 and 6, and #8 – 10. p.V87_N89del (c.259_267del) and p.L47P (c.140 T > C) heterozygote was reported to be pathogenic (Jung et al., 2018; Shin et al., 2019). p.G307V (c.920G > T) heterozygote mutation has not been reported to be pathogenic but segregated in the patient and siblings.

*F* female, *M* male, *Rt* right, *Lt* left, *vHIT* video head impulse test, *oVEMP* ocular vestibular evoked myogenic potential, *cVEMP* cervical vestibular evoked myogenic potential, *NL* normal, *AbNL* abnormal, *ASCC* anterior semicircular canal, *LSCC* lateral semicircular canal, *PSCC* posterior semicircular canal, *N/A* not applicable because the test was not conduct.

^†^Pure tone threshold was calculated by the average threshold of 250, 500, 1000, 2000 Hz.

## Discussion

The most notable findings of this study can be summarized into four points. First, baseline vestibular function was normal and function was decreased after excessive mechanical stimulation (hypergravity stimulation) in all the studied genotypes of mice. Second, the vestibular function decrease was more significant in the *Kcnq4*^p.W277S/p.W277S^ mice than in the *Kcnq4*^+/+^ and *Kcnq4*^+/p.W277S^ mice after stimulation. From the first and second points, we can assume that hypergravity stimulation can be used to evaluate the vulnerability of vestibular function to mechanical stimulation. Third, the decreased vestibular function was likely caused by the loss of vestibular hair cells. Fourth, vestibular dysfunction was also present in human patients with *KCNQ4* mutations.

An animal experiment using *Kcnq4* knockout mice showed minimally decreased basal vestibular function^19^. In our study, we could not find any difference in basal vestibular function among genotypes. It can be assumed that total loss of function in *Kcnq4* knockout mice might show a more severe phenotype than partial loss of function due to the p.W277S mutation. In addition, in the former study, the age of the mice used for the VOR measurement was not indicated; therefore, a direct comparison of the knockout and mutant mice could not be definitively determined. We used relatively young mice (8–12 weeks), in which vestibular function might not have been deteriorated because vestibular function is expected to decline with age because mechanical challenges are expected to accumulate. Therefore, hypergravity stimulation seems to be a good method for identifying vestibular organ vulnerability according to vestibular pathology. This new method can be used to study the vestibular system as a noise stimulation used in the study of the cochlear system.

Hypergravity stimulation induced a gain reduction at a stimulation frequency of 0.32 – 1.28 Hz and a phase difference increase at stimulation frequencies of 0.16—0.64 Hz in the SHA test, a reduction in Tc in the SV test, and a reduction in modulation in the OVAR test in all the studied genotypes. Asymmetry showed a tendency to increase, but the difference was not statistically significant, which was likely to result from bilateral partial VOR impairment. The vectorial direction of hypergravity stimulation in this study was more likely to affect saccular function, which detects Earth’s vertical linear acceleration and gravity. Therefore, the stimulation to the semicircular canals and the utricle is expected to be weaker. However, the parameters in the test showed decreased lateral semicircular canal function, as shown by the gain and Tc decrease. This might have resulted from head movement of the mice during stimulation. The mouse body and head were not fixed in the cage, and the orientation of the head could be continuously changed during stimulation by head and body movement, which was observed during stimulation by a camera installed in the cage (Supplementary Video S1). As a result, the axis of the semicircular canal can be frequently aligned to the vectorial direction to which the acceleration stimulation was applied, which could cause semicircular canal dysfunction. To prevent head movement of mice during the stimulation, the head should be restrained for 24 h, but this inevitably causes distress to the mice and limits food and water intake. Therefore, we did not restrain the mice during the stimulation. The gain reduction at 0.32—1.28 Hz and the reduction in Tc suggest that the higher frequency area (> 0.3 Hz) of the lateral semicircular canal was mainly affected by the stimulation. The response to high-frequency acceleration stimulation is induced mainly by the depolarization of type 1 hair cells where calyx nerve endings are distributed, which is supported by the direct nerve recording from vestibular nerve during rotation and galvanic stimulation and presence of more calcium-activated potassium channels and voltage-activated potassium channels^23–25^. Hence, it can be assumed that the parameter changes observed in the SHA and SV tests mainly resulted from injury to type 1 hair cells and/or distributed nerves where KCNQ4 is located, which was demonstrated in the immunofluorescent staining of each genotype. The assumption that misaligned vectorial stimulation of certain vestibular organs could induce vestibular loss by head or body movement could not be applicable to the findings in the utricle. The decrease in the ratio of intact type 1 hair cells with KCNQ4 and calretinin-positive calyx were significant in the saccule, but not in the utricle. Utricular hair cells detect earth horizontal linear acceleration and roll plane movement against gravity. Hair cells are arranged multidirectionally from the curvilinear structure of the striola in the otolithic organs, which enables hair cells to be depolarized by linear acceleration from various directions in the utricle and saccule. This feature is more complex than those in semicircular canals. The saccule mainly detects Earth’s vertical linear acceleration, and it can be assumed that hypergravity stimulation in the experiment, which was similar to the vertical stimulation applied by Earth’s gravity, might mainly affect saccular hair cells. In contrast, it is tempting to speculate that utricular hair cells might have been less affected by the stimulation because the stimulation was likely to be weaker due to the different vectorial axes of the stimulation, even with the head movement during the stimulation, and because the stimulation might be dispersed throughout the utricular hair cells due to their multidirectional distribution. In the OVAR tests, which evaluate the function of otolithic organs, only modulation was significantly decreased after stimulation in all the genotypes. Although the definitive origin of the modulation and bias component has not been fully identified, it can be speculated that the saccule is more involved in the parameters in OVAR test, especially modulation.

Most of the VOR parameters were more significantly decreased in the *Kcnq4*^p.W277S/p.W277S^ mice compared to the *Kcnq4*^+/+^ and *Kcnq4*^+/p.W277S^ mice after stimulation. A significantly lower gain at the stimulation frequency of > 0.32 Hz and Tc support the possibility of a larger loss of type 1 hair cells or calyx nerve endings distributed to the cells in the semicircular canal ampullary sensory epithelium in the *Kcnq4*^p.W277S/p.W277S^ mice. The modulation was significantly lower in the *Kcnq4*^p.W277S/p.W277S^ mice than in the *Kcnq4*^+/+^ and *Kcnq4*^+/p.W277S^ mice, but the bias was not different among the genotypes after stimulation. It is generally thought that the velocity storage mechanism of the central nervous system and the linear VOR orientation mechanism are responsible for the bias and modulation components, respectively^26–28^. Therefore, the modulation component was more likely to be affected by the different degrees of saccular dysfunction as described above, and the bias decrease was not significantly different among the genotypes. However, the signal to the velocity storage center from peripheral vestibular organs might be decreased after stimulation, which should decrease the bias value. If there were different degrees of vestibular loss in the otolithic organs among the genotypes, the bias value was likely to be different according to the genotypes. It can be assumed that the resolution of bias was not sensitive enough to reflect the certain degree of injury in the otolithic organ in the current experimental condition or that hypergravity stimulation might strongly influence the central velocity storage system equally regardless of the genotypes.

**Figure 9:**
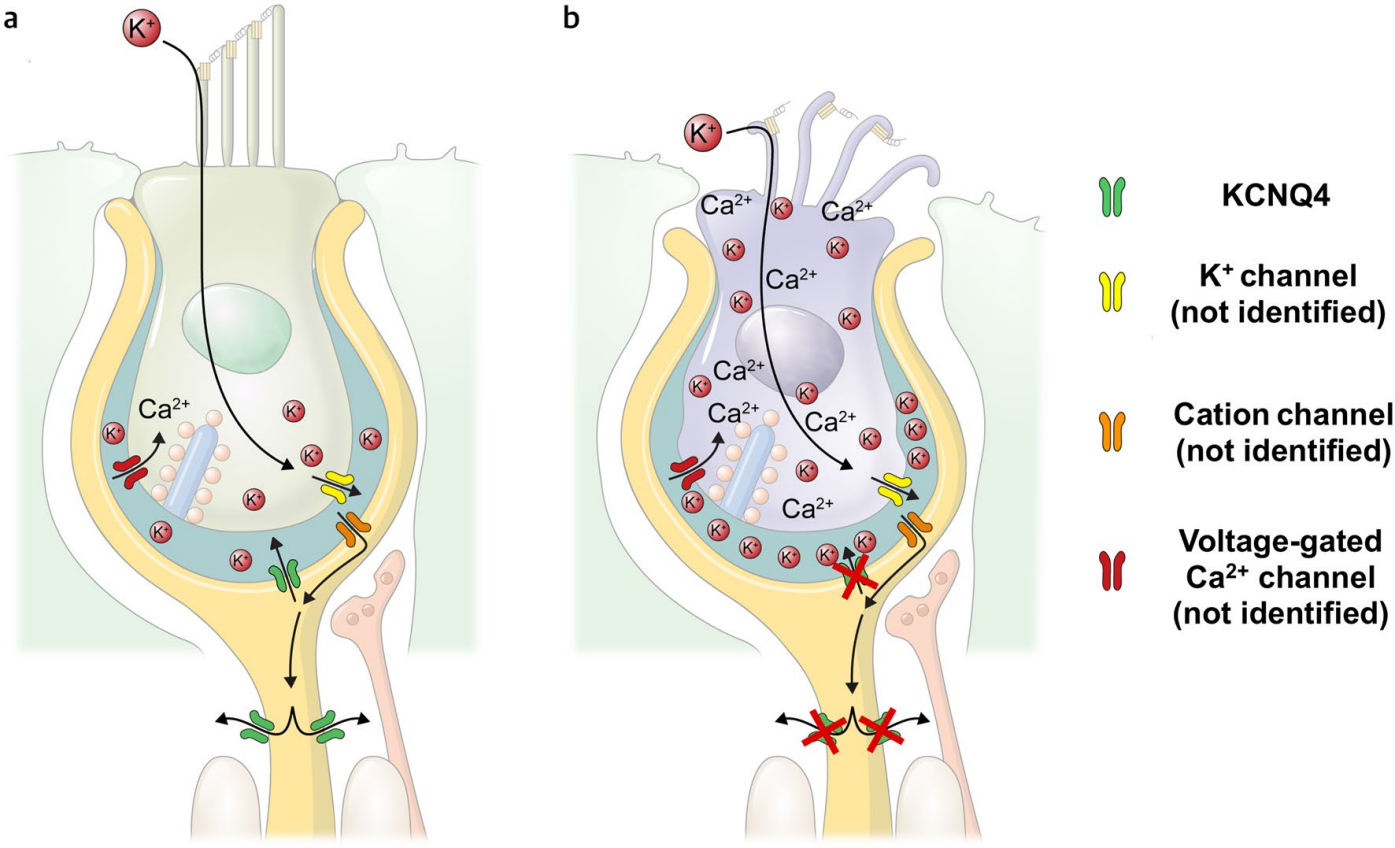
Schematic drawing of vestibular hair cell damage in KCNQ4 dysfunction. (**a**) Type 1 vestibular hair cells with normal KCNQ4. One of the K^+^ channels excreting K^+^ from the vestibular nerve is KCNQ4, which is located on the inner surface of the calyx and on the heminode. (**b**) Type 1 vestibular hair cells with KCNQ4 dysfunction. KCNQ4 dysfunction can cause the accumulation of K^+^ in the synaptic cleft, which leads to decreased K^+^ excretion from hair cells. This ultimately causes cation toxicity in hair cells through the intracellular accumulation of K^+^ and Ca^2+^.

The more severe vestibular dysfunction in the *Kcnq4*^p.W277S/p.W277S^ mice can be explained by two possible mechanisms. The first is the possibility of more severe hair cell loss, and the other is the possibility of vestibular nerve injury, including calyx nerve ending injury, since KCNQ4 is located at the inner apical surface of the calyx and heminode of the calyx-only afferent nerve. We examined the ratio of intact hair cell to total KCNQ4 and calretinin-positive stain calyx count and the number of KCNQ4-positive calyx nerve endings using immunofluorescent staining to determine which mechanism was more likely to contribute to vestibular dysfunction after hypergravity stimulation. The ratio of intact hair cell to total KCNQ4 and calretinin-positive stain calyx count was significantly decreased in the anterior and lateral semicircular canal ampulla and saccule of the *Kcnq4*^p.W277S/p.W277S^ mice than those of the *Kcnq4*^+/+^ and *Kcnq4*^+/p.W277S^ mice, but the number of KCNQ4-positive calyx nerve endings in the vestibular sensory epithelium was not significantly different among the genotypes. Although we could not evaluate the function of the anterior semicircular canal in the experimental setting, canal function should be decreased based on the histological findings. Based on the above findings, we speculate that the difference in vestibular dysfunction among the genotypes after stimulation was more likely to be caused by hair cell loss than calyx and nerve damage. K^+^ ions entering type 1 vestibular hair cells escape from the cytosol through K^+^ channels on the basolateral surface. Several studies have investigated the presence of K^+^ channels at the basolateral surface of vestibular hair cells, but the molecular identity of the channel has not been fully revealed^3,16,23^. After exiting the cell, the K^+^ ions stay in the synaptic cleft between the hair cell and calyx, then enter the calyx through cation channels. The ion channel has not been fully identified, and a cyclic nucleotide-gated channel was recently suggested as one of them^29^. The K^+^ ions in the calyx and nerve escaped from the cytosol through K^+^ channels at the cellular membrane, one of which is likely to be KCNQ4 on the inner surface of the calyx and on the heminode. If this is not accomplished, K^+^ ions cannot enter the calyx and nerve appropriately, and the [K^+^] in the synaptic cleft increases. Then, the increased [K^+^] in the synaptic cleft interferes with the efflux of K^+^ ions from type 1 vestibular hair cells, and intracellular [K^+^] and [Ca^2+^] increase, which may consequently cause prolonged depolarization and cellular damage (Fig. 9).

Since KCNQ4 is distributed only in the vestibular neuron innervated to type 1 hair cells, if the depolarization duration was increased in the cells, the mean Tc value of [Ca^2+^] decay in the cells distributed in the central and peristriolar regions should be increased even though the supporting cells and few type 2 hair cells were included in the analysis. In the experiment, the Tcs in the *Kcnq4*^+/p.W277S^ and *Kcnq4*^p.W277S/p.W277S^ mice were significantly increased compared with that in the *Kcnq4*^+/+^ mice, and this finding is likely to support the above hypothesis for hair cell loss. It is not clear why the damage was confined only to hair cells and not to calyx where KCNQ4 is distributed. It was reported that K^+^ channels other than KCNQ4, such as K_2p,_ K_v_1, K_v_7.2, K_v_7.3, and K_v_7.5, are located in calyx-type vestibular neurons by immunohistochemistry^15,16^. An electrophysiological experiment also indicated that K_v_1 and K_v_7 play a role in the efflux of K^+^ ions from calyx-type vestibular neurons^2^. Based on the findings of those studies, several K^+^ channels in addition to KCNQ4 may be involved in K^+^ efflux in calyx-type vestibular neurons. K^+^ ion effluxes from neurons can be partially compensated by the other K^+^ channels, but hair cells may be more vulnerable to damage by increased intracellular [K^+^] even though it is partially compensated by the activity of the other K^+^ channels in the vestibular nerve. Furthermore, there was no K^+^ ion movement by diffusion from the synaptic cleft to the extracalyceal space, as reported by Spaiardi et al., which could an additional factor in hair cell damage ^2^. Therefore, [K^+^] in the synaptic cleft increased during stimulation, which might consequently cause damage to hair cells earlier than vestibular neurons.

The vestibular dysfunction was milder in the *Kcnq4*^+/p.W277S^ mice than that in the *Kcnq4*^p.W277S/p.W277S^ mice . VOR gain values in SHA test seemed to have decreasing tendency when it compared to *Kcnq4*^+/+^ mice, but the gain value was significantly decreased only at 0.32 Hz stimulation frequency. Also, the ratio of intact hair cell decrease was only significant in saccule. In contrast, cochlear phenotype in *Kcnq4*^+/p.W277S^ mice is nearly as severe as *Kcnq4*^p.W277S/p.W277S^ mice^30^. Human vestibular phenotype in heterozygote mutation also seemed to be more prominent than that in *Kcnq4*^+/p.W277S^ mice when considering the 7 of 10 patients with heterozygous mutations had a certain amount of vestibular dysfunction in clinical vestibular tests in this study. The different phenotype between cochlear and vestibule in the p.W277S mutation mice might be originated from the different vulnerability to the mechanical stimulation to each cochlea and vestibule. For the different vestibular phenotypes between the mutational mouse and human has four possible explanations. First, the stimulation type between humans and mice was different. The mice were stimulated with 6 g for 24 h, but the patients were likely to be exposed to cumulative chronic, recurrent, and sometimes excessive stimulation for a long time. Therefore, the degree and extent of vestibular loss between the patients and the mice could be different. Second, the mutation types were different, which might lead to different degrees of KCNQ4 dysfunction. Third, the vulnerability of vestibular organs can be different between mice and humans. Fourth, the test used in human and mutational mice was different. In human, the semicircular canal function was evaluated with vHIT that was a higher frequency stimulation range in the evaluation of vestibular function than rotation test used in the mouse. Also, otolithic organ responses to sound stimulation (VEMPs) and off-vertical axis rotation could be different. Although there is little discrepancy between the human and mouse vestibular phenotypes in the presence of *KCNQ4* mutations, it is clear that *KCNQ4* mutations can cause vestibular dysfunction.

It was possible that KCNQ4 dysfunction in the central vestibular system may have some contribution to the vestibular dysfunction in *Kcnq4*^p.W277S/p.W277S^ mice. It was reported that KCNQ4 expressed in central auditory pathway^31^, but its expression in central vestibular pathway has not been identified. If KCNQ4 also exists in central vestibular pathway, it can contribute to the vestibular dysfunction directly or by the failure in the compensating mechanism. The existence and the role of KCNQ4 in the central vestibular system should be investigated in the future study.

This is the first study to identify vestibular dysfunction caused by mechanical stimulation using an animal model of *Kcnq4* mutations as well as vestibular dysfunction in patients with *KCNQ4* mutations. The findings of this study suggest that KCNQ4 plays an essential role in preserving vestibular function against excessive mechanical stimulation. We believe that the results of this study can contribute to elucidating the role of KCNQ4 in the vestibular system.

## Methods

### Study approval

All of the animal experimental procedures and a study enrolling humans were approved by the Yonsei University Health System Institutional Animal Care and Use Committee (approval No. 2016-0254) and Institutional Review Board of Severance Hospital, Yonsei University College of Medicine (approval No. 2017-0295). All the animal experiments were performed in accordance with ARRIVE 2.0 guidelines and regulations. Before genetic study and vestibular function measurement in patients with *KCNQ4* mutations, informed consent was obtained from all participants and the studies were performed in accordance with the Declaration of Helsinki. All the methods in this study were performed in accordance with the relevant guidelines and regulations.

### Creation of the Kcnq4 ^p.W277S/p.W277S^ mice

A p.W277S/p.W277S *Kcnq4* knock-in mouse model was generated by Macrogen Inc. (Seoul, Korea) using a CRISPR/Cas9 technique, as previously reported (Wang et al., 2016). The 830G>C mutation in exon 5 was applied to p.W277S/p.W277S *Kcnq4* transgenic mice. The transgenic mouse model was established with a CRISPR/Cas9 system. Two 23 nt guide RNAs, 5’- CCTCCTATGCCGACTCGCTCTGG -3’ and 5’- ATGCCGACTCGCTCTGGTGGGGG -3’, were designed to drive Cas9 to the target exon 5 of the *Kcnq4* gene, where mutation 830G>C is located.

Genotype sequencing was performed with sense primer 5’- AGGCTGGAAAGGCGATG -3’, anti-sense primer 5’- CGGTACACATCACAAGGGCT -3’, restriction enzyme NdeI, and a silent mutation for preventing recut of the knock-in allele by single guide RNA (size of amplicons: wild-type: 1,291 bp, knock-in: 656 bp + 635 bp). The background of the *Kcnq4*^p.W277S/p.W277S^ mice was C57BL6/J, but in the embryo transfer procedure, the mouse background was mixed with C57BL6/N (Orient Bio, Seoul, Republic of Korea). The *Kcnq4*^+/p.W277S^ mice were made by cross-breeding *Kcnq4*^+/+^ (C57BL6/N) and *Kcnq4*^p.W277S/p.W277S^ mice. All the experiments were conducted using 8- to 12-week-old mice.

### Hypergravity stimulation

Before hypergravity stimulation, preparation for vestibulo-ocular reflex (VOR) measurement was performed. A skin incision approximately 1 cm in length was made anteriorly from the vertex, and a small metal nut with a screw hole for the fixation of the head to the head holder in the animal rotator was anchored to the vertex using dental cement. We waited 2-3 days for the stabilization of the head holder. Four mice of each genotype were placed in a chamber of an eccentric animal accelerator (Supplementary Video S2). We administered 6-*g* stimulation for 6-24 hours using the custom default program of the motor controller. The floor of cage was positioned to be vertical to the centrifugal force during the rotation, therefore hypergravity forces were applied to mice, which was already described and used in the previous reports^32,33^. During the stimulation, the mice were provided with the same feed and water as they had in their cage. All the mice used in the experiments were tolerant to the stimulation, and no mouse was found to be dead after the stimulation.

### Retigabine injection

For the evaluation of the effect of retigabine (Sigma-Aldrich, St. Louis, MO, USA) on the mouse vestibular organ against hypergravity stimulation, retigabine was diluted in dimethyl sulfoxide (DMSO) (50 mg/ml, Sigma-Aldrich), and a final dose of 10 μg/g was injected i.p. to the *Kcnq4*^+/+^ mice at 24 hours and 2 hours before stimulation. The same volume of DMSO was injected i.p. to the control mice instead of retigabine before stimulation at the same time point.

### Measurement of mouse vestibular function

The vestibular function of the mice before and after hypergravity stimulation was evaluated by measuring the VOR in the animal rotator in dark (Supplementary Fig. S3). The evaluation of vestibular function using the VOR with whole-body rotation has been popularly used in animal and human subjects. Stimulation of vestibular organs by angular acceleration induces eye movement in the opposite direction of the stimulation to provide stable vision^34^. The SHA and the SV tests measured the VOR from the lateral semicircular canal, and the OVAR test measured the VOR from otolithic organs. If a subject has vestibular weakness in those compartments of the vestibular system, the VOR is impaired; hence, the gain, Tc, modulation, and bias decrease, and the phase difference and asymmetry increase. The results of the SHA and SV tests represent lateral semicircular canal function, and the result of the OVAR test represents otolithic organ function. Before the experiment, the mouse was anesthetized with isoflurane gas (2-4%), and triangular paper markers (each side = 0.4 mm) for eye tracking were attached to the center of the eyeball using an adhesive. Then, the mouse was placed in a cylindrical restrainer connected to the vestibular turntable. The interval between the end of hypergravity stimulation and the VOR measurement was 1.5–2 hours. The mouse’s head was fixed to the restrainer using the previously implanted head fixation anchor in the scalp. The VOR was measured after the mouse was completely awaken from the anesthesia. Three methods of angular acceleration stimulation were applied for the measurement. Those were the SHA stimuli with stimulation frequencies of 0.08, 0.1, 0.16, 0.32, 0.64, and 1.28 Hz, the SV stimulation with acceleration of 3000°/s^2^ and 150°/s to clockwise and counterclockwise directions, and the OVAR for which the turn table of the rotator was tilted 30° from the vertical axis and the SV rotation stimulus was applied for 30 s to clockwise and counterclockwise directions. The VOR parameters in each stimulation condition were gain, phase, and symmetry for the SHA test, time constant (Tc) for the SV test, and modulation and bias for the OVAR test. The definition of each parameter of the SHA and SV tests was as follows.

*Gain* = slow phase peak eye velocity induced by rotation divided by peak rotation velocity.

*Phase* = phase difference between the time point of peak eye velocity and peak rotation velocity.

*Symmetry* = (peak eye velocity to right direction – left direction/peak eye velocity to right direction + left direction) X 100.

*Tc* = time point at which the slow phase velocity of nystagmus was reduced to 63% from the peak slow phase velocity of nystagmus during and after the step.

In the OVAR test, the parameters of the slow phase eye movements induced in each horizontal, vertical, and torsional plane were extracted by least square fitting a sinusoid (modulation) plus off set (bias) on the value of slow phase velocity and position. The modulation and bias were then calculated on the velocity and the position ocular traces in each plane^35^. The modulation and bias in vertical eye movements were consistent and significant in the experiment; therefore, the results of vertical eye movement were adopted in this study.

### Immunohistochemistry and cell counting

The mice were anesthetized with tribromoethanol (16 ml/kg) via intraperitoneal injection and euthanized by decapitation. The mice with stimulation were euthanized at 1.5 – 2 hours after stimulation. Temporal bones were removed from the mice, and the membranous vestibular labyrinth was isolated by meticulous dissection in perilymph-like solution (150 mM NaCl, 3.6 mM KCl, 1 mM MgCl_2_, 0.7 mM CaCl_2_, 5 mM glucose, and 10 mM HEPES, pH 7.4). For cryosection, the isolated vestibular end-organs were fixed with 4% paraformaldehyde (PFA) for 20 min, and the tissues were rinsed in order with 10%, 20%, and 30% sucrose solution followed by incubation with 30% sucrose solution for 15 min. Then, specimens were immersed in 15% sucrose and OCT compound (4583, Sakura Finetek USA, Inc., Torrance, CA, USA) and incubated overnight at 4°C. The solution was changed to OCT compound and stored at room temperature. The tissue was embedded in OCT compound, frozen, and sectioned to 10 µm using a cryotome (Microtome Cryostat Microm HM 525, Thermo Fisher Scientific, Waltham, MA USA). For whole mounts, each utricle, ampulla, and saccule was isolated and fixed with 4% PFA for 20 min. The posterior semicircular canal ampulla was not included in this study because the tissue must be prepared as a separate tissue, which results in damage during dissection. Furthermore, the prepared tissue was easily washed out from the slide during the washing procedure. The tissues were decalcified with 10% ethylenediaminetetraacetic acid solution to remove bone chips and otoliths. The prepared tissues were permeabilized and blocked in phosphate-buffered saline (PBS) with 0.2% Triton X-100 (PBS-TX) and 5% bovine serum albumin (BSA) at room temperature. They were then washed three times with PBS-TX and incubated overnight at 4°C with an anti-KCNQ4 mouse monoclonal antibody (1:100, ab84820, Abcam, Cambridge, UK) and anti-calretinin rabbit polyclonal antibody (1:150, ab702, Abcam) in PBS-TX with 2.5% BSA. These cells were then washed five times with PBS-TX and Alexa-568-goat anti-mouse antibody (1:1000, A11004, Invitrogen), Alexa-647-donkey anti-rabbit antibody (1:1000, A31573, Invitrogen), FITC-conjugated phalloidin (1:200, P5282, Sigma-Aldrich), and DAPI (1:10000, D1306, Thermo Fisher Scientific) in PBS-TX with 2.5% BSA at room temperature. These were then washed five times with PBS-TX. After cryosection and whole-mount preparation, the tissues were placed on slides and covered with coverslips with Fluoromount Aqueous Mounting Medium (F4680, Sigma, St. Louis, MO, USA). The prepared tissues were imaged using a Zeiss LSM 780 or 980 confocal microscope (Carl Zeiss, Jena, Germany). The ratio of hair cell count with intact cilia to total hair cell count in which KCNQ4 and calretinin-positive stain was counted manually in mice with and without stimulation. (Supplementary Fig. S4). The cell count was performed with ZEN software (Carl Zeiss).

### TEM and immunogold staining

*Kcnq4*^+/+^ mice were anesthetized with tribromoethanol (16 ml/kg) via intraperitoneal injection. Transcardiac perfusion with PBS followed by 4% PFA was done. After the cardiac perfusion, temporal bone was harvested and fixed with 4% PFA overnight in room temperature with a punch hole made at the apex of cochlea. Then, vestibular saccule and utricle were harvested meticulously with fine forceps. The harvested tissues were kept in 125mM EDTA for 30 minutes in room temperature. After that, blocking was performed in 5% BSA for 1 hours in room temperature and the tissues were washed 3 times for 10 minutes with Tris-buffered saline (TBS). Anti-KCNQ4 mouse monoclonal antibody (1:500, Abcam) was applied for 3 hours in room temperature and then the tissues were washed 3 times by 10 minutes with TBS. Anti-Mouse IgG (whole molecule)–Gold antibody produced in goat (1:20, 10nm gold particle, G7652-.4ML, MERCK, USA) was applied for overnight in room temperature and the tissues were washed 3 times by 10 minute with TBS. The tissues were fixed for 12 hours in 2% Glutaraldehyde - 2% PFA in 0.1M PBS (pH 7.4) and then, washed in 0.1M PBS. The tissues were post-fixed with 1% OsO4 in 0.1M PBS for 2 hours and dehydrated with an ascending ethanol series (50, 60, 70, 80, 90, 95, and 100%) for 10 minutes each and infiltrated with propylene oxide for 10 minutes. The tissues were embedded with a Poly/Bed 812 kit (Polysciences, Warrington, PA, USA) and polymerized in an electron microscope oven (TD-700, DOSAKA, Japan) at 65℃ for 12hr. The block was placed in Ultra-microtome (UC7, Leica Microsystems, Vienna, Austria) equipped with a diamond knife, and was cut into 200nm semi–thin section and stained toluidine blue for the observation of optical microscope. The region of interest was then cut into 80nm thin sections using the ultra-microtome, placed on copper grids, double stained with 5% uranyl acetate for 20min and 3% lead citrate for 7min. Imaging was performed with a TEM (HT7800, HITACHI, Tokyo, Japan) at the acceleration voltage of 80kV equipped with a RC camera.

### Intracellular [Ca2 +] live imaging in the sensory epithelium of the utricle and ampulla

Ca^2+^ influx into hair cells follows intracellular K^+^ influx, which induces the release of synaptic vesicles to the synaptic cleft for signal transduction in vestibular neurons and is then excreted from the cell during repolarization^36^. We tried to measure the Tc of intracellular [Ca^2+^] to identify if the depolarization duration was prolonged in the *Kcnq4*^p.W277S/p.W277S^ and *Kcnq4*^+/p.W277S^ mice as an indirect method. Membranous labyrinth of the vestibular organ was carefully harvested as described above. The utricle and ampulla were sectioned from the tissue and otoconia, and the roof epithelium was carefully removed by microdissection. The tissue was incubated with 5 μM Fluo-4 AM (Thermo Fisher Scientific) in Ca^2+^-free solution (150 mM NaCl, 3.6 mM KCl, 1 mM MgCl_2_, 10 mM HEPES, 0.5 mM EGTA) for 1 hour. Then, the utricle and ampulla were placed on the perfusion chamber under a confocal microscope (LSM 780, Carl Zeiss) and held with a three-axis hanging Joystick oil hydraulic micromanipulator (Narishige, Minamikarasuyama 4-chome, Setagaya-ku, Tokyo, Japan). The chamber was perfused with Ca^2+^-free solution for 3 minutes, and then, perilymph-like solution was perfused for 1 minutes followed by Ca^2+^-free solution perfusion. The solutions were heated at 36.5°C during perfusion. The fluorescent signal change from the sensory epithelium was recorded at 5-second intervals until the signal decreased to the point at which no more change was detected. We could not identify the cell type in the vestibular sensory epithelium during live imaging *ex vivo*; therefore, we tried to measure Ca^2+^ signaling from the cells of the central and peristriolar regions. Each signal from a focused individual cell was captured by the region of interest tool in ZEN software. The signal was measured in central and peristriolar area of ampullary and macular epithelium. The background signal of each region was subtracted from the fluorescent signal, and the fluorescent intensity change was analyzed. The signal decrease showed a negative exponential curve; therefore, we calculated Tc by curve fitting using the formula depending on exponential pattern (single or double) below with Origin Pro 2020 (OriginLab Software, Northampton, MA, USA). We selected Tc value with higher R square value as data.

*y* = *y*_0_ + *A*_1_*e*^−*x/t1*^ (*y*_0_, basal value after decay; *A*_1_, amplitude; *e*, exponential constant; *t*^*1*^: time constant).

*y* = *y*_0_ + *A*_1_*e*^−*x/t1*^ + *A*_*2*_*e*^−*x/21*^ (*y*_0_, basal value after decay; *A*_1_ and *A*_2_, amplitudes; *e*, exponential constant; *t*_*1*_ and *t*_*2*_: time constants).

### Measurement of vestibular function in patients with KCNQ4 mutation

We enrolled patients with *KCNQ4* mutations identified by next-generation sequencing or whole-exome sequencing. All the patients had bilateral sensorineural hearing loss and family histories of hearing loss with autosomal dominant inheritance patterns. Vestibular function was measured by video head impulse test (vHIT) (ICS Impulse, Otometrics, Taastrup, Denmark) for each semicircular canal and ocular and cervical vestibular evoked myogenic potentials (oVEMP and cVEMP) for utricular and saccular functions. The vHIT was conducted 20 times in random directions for each semicircular canal, with a peak velocity of 200 to 250 degrees per second, rotation amplitude of ~15 degrees, and duration of 150 to 200 msec as described in the literature^37^. The VOR gain was calculated automatically. The parameters for abnormal vestibular function in the test were gain below the normal value presented by the company and the presence of catch-up saccade. For the VEMP responses, electromyographic changes in the ipsilateral sternocleidomastoid muscle (cVEMP) or the contralateral inferior oblique ocular muscle (oVEMP) by 500 Hz 95 dB HL tone burst stimulation were recorded with an Audera system (GSI, CA, USA) following the same protocol in the literature^38^. We regarded the response as abnormal if there was no response after the sound stimulation.

### Experimental design and statistics

If the expected difference of gain value and intact hair cell proportion after hypergravity stimulation among *Kcnq4*^+/+^, *Kcnq4*^+/p.W277S^, and *Kcnq4*^p.W277S/p.W277S^ mice were 0.2 or more, achieving a power value of 0.999 and alpha value of 0.05 required that number of mouse in each group was 12 and 14 for ANOVA and t-test. Therefore, we initially planned to perform the experiments using 12 mice per group. However, we were able to get statistically significant results less than 12 mostly. In each dataset, normality and equal variance were evaluated by the Shapiro-Wilk test and Brown-Forsythe test. The differences in gain/phase/symmetry in the stimulation frequencies in the SHA test, Tc in the SV test, and modulation and bias in the OVAR test before and after stimulation among genotypes or between the mice treated with DMSO and retigabine or different stimulation durations were evaluated by two-way repeated-measures ANOVA with Holm-Sidak’s posttest. The difference in the number of hair cells among the genotypes with or without acceleration stimulation was evaluated by two-way ANOVA with Holm-Sidak’s posttest. The differences in the number of hair cells between the mice treated with DMSO or retigabine were evaluated by the t-test or Mann-Whitney rank sum test. *p* < 0.05 was considered to be significant. Statistical analysis was performed with SigmaPlot 13.0 (Systat Software, San Jose, CA, USA). Biological replicates were used for the analysis. The values presented in this study are the mean ± SD in data with a normal distribution and the median (interquartile range) in data without a normal distribution. The authors declare that the data supporting the findings of this study are available within the paper and its supplementary information files.

### Supplementary Information

Supplementary Information 1.

Supplementary Information 2.

Supplementary Information 3.

Supplementary Information 4.

Supplementary Information 5.

Supplementary Figure S1.

Supplementary Figure S2.

Supplementary Figure S3.

Supplementary Figure S4.

Supplementary Video 1.

Supplementary Video 2.

## Data Availability

The datasets for this study can be found in the Supplementary Materials
