# Supplementary figures and images for: Vestibular hair cells are more prone to damage by excessive acceleration insult in the mouse with KCNQ4 dysfunction
Source: Sci Rep. 2024 Jul 3;14:15260. doi: 10.1038/s41598-024-66115-9 (PMC11219875; doi:10.1038/s41598-024-66115-9)

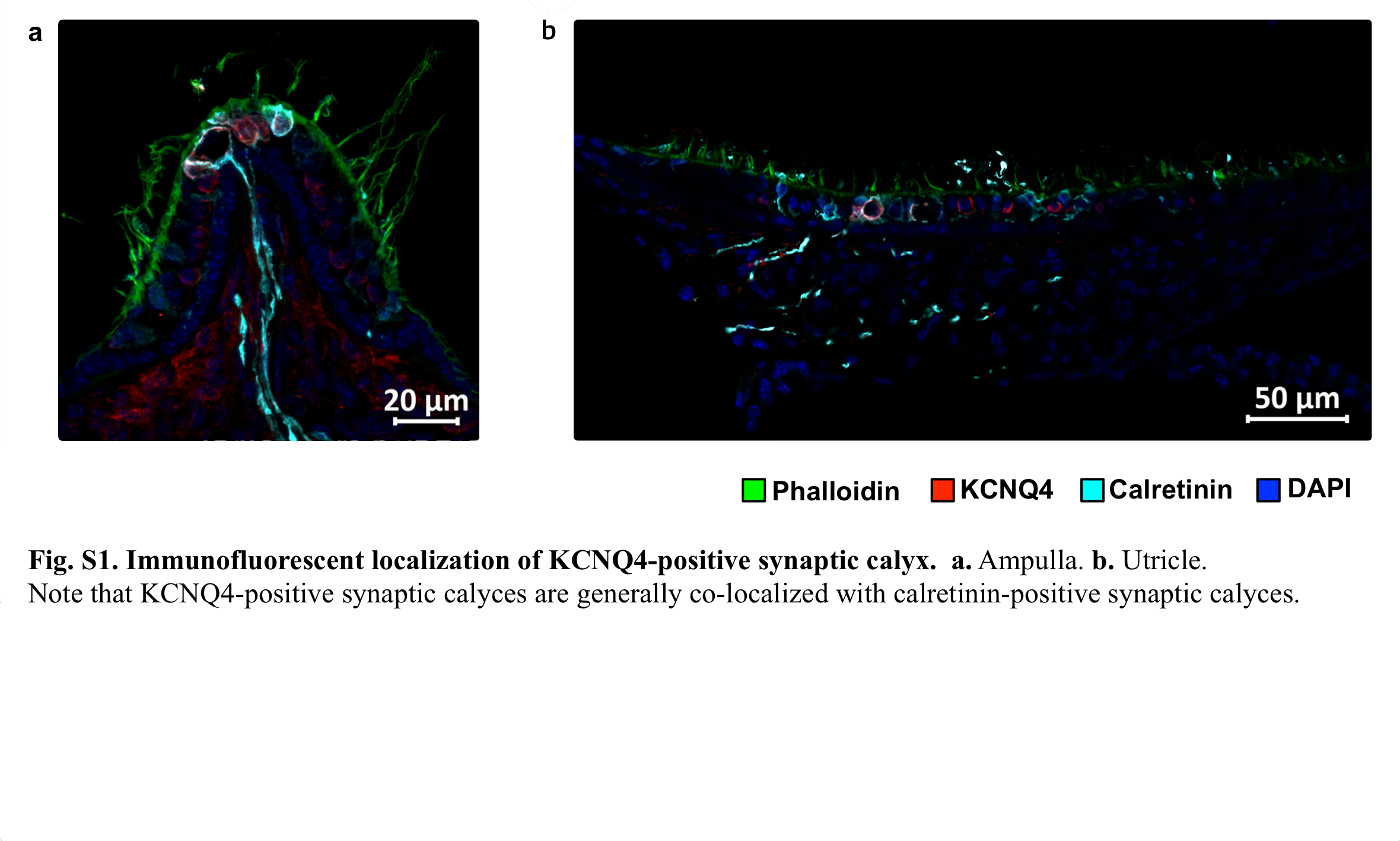

Supplement: Supplementary file 6 — Supplementary Figure S1. [file 41598_2024_66115_MOESM6_ESM.tiff]

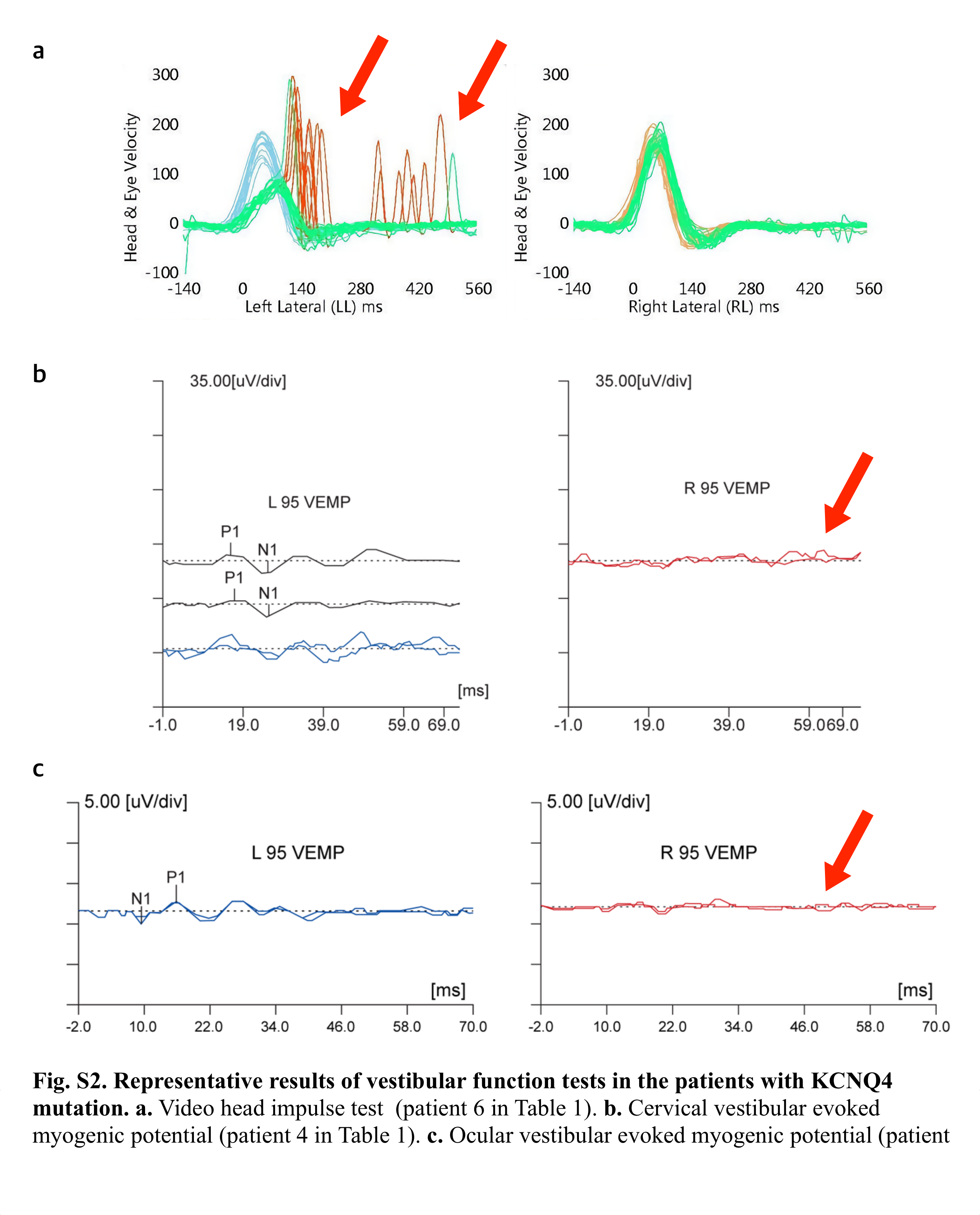

Supplement: Supplementary file 7 — Supplementary Figure S2. [file 41598_2024_66115_MOESM7_ESM.tiff]

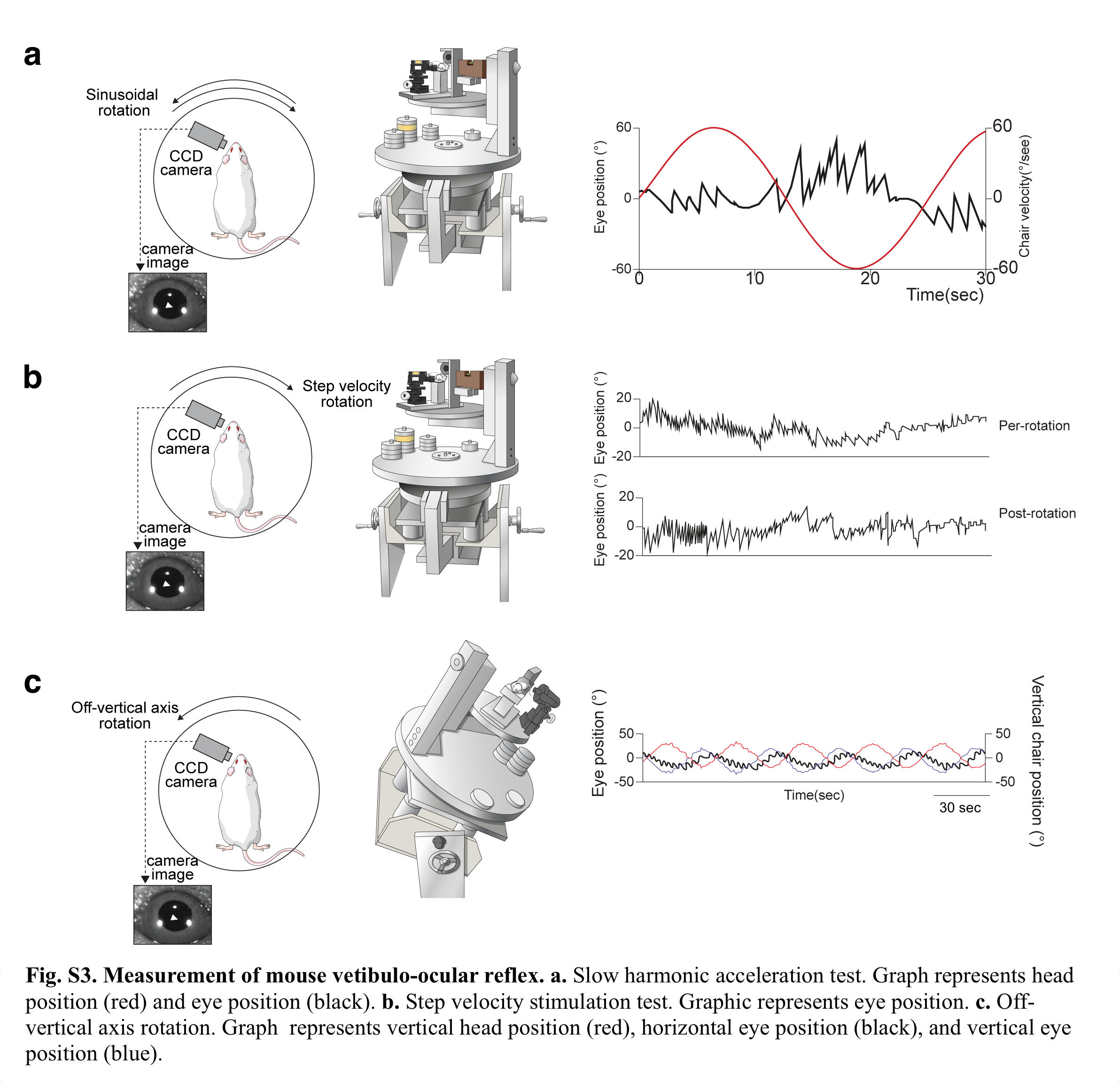

Supplement: Supplementary file 8 — Supplementary Figure S3. [file 41598_2024_66115_MOESM8_ESM.tiff]

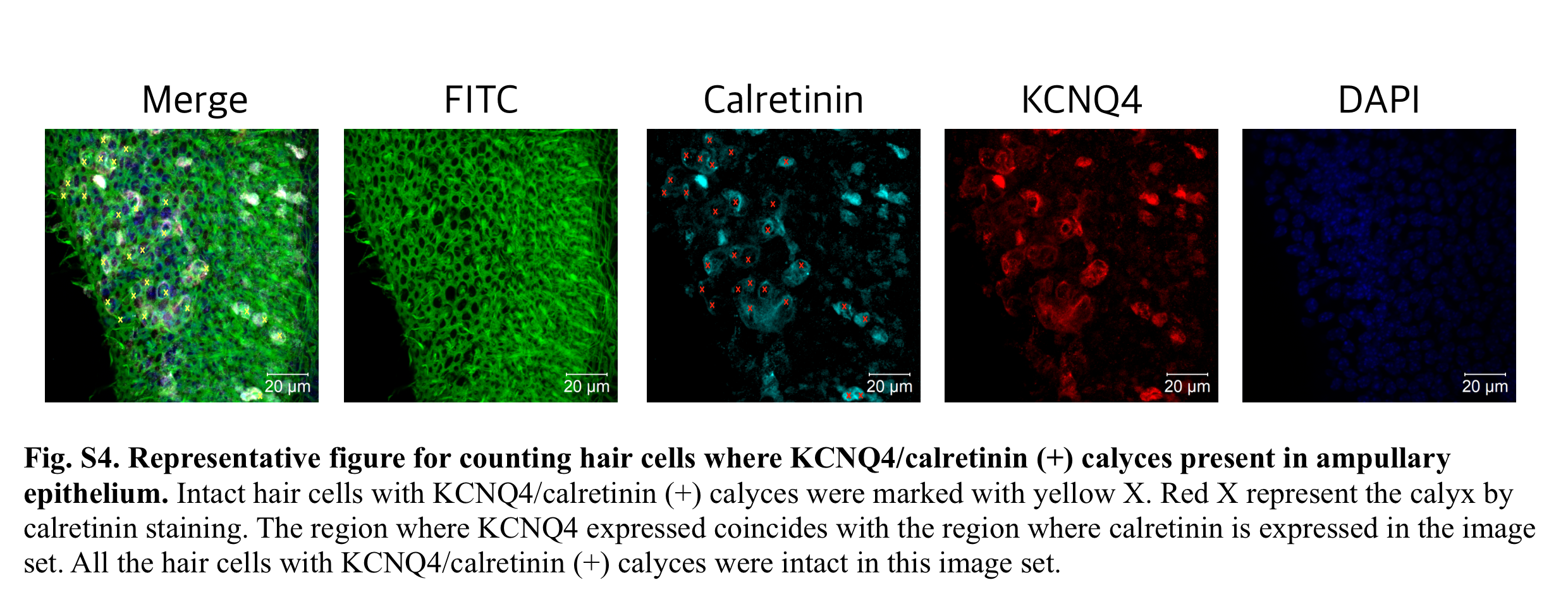

Supplement: Supplementary file 9 — Supplementary Figure S4. [file 41598_2024_66115_MOESM9_ESM.tiff]
